# Supplementary material for: Prevalence and determinants of underweight, overweight, and obesity among reproductive-aged Bangladeshi women: Evidence from Bangladesh Demographic and Health Survey 2022
Source: PLoS One. 2026 Feb 25;21(2):e0341037. doi: 10.1371/journal.pone.0341037 (PMC12935259; doi:10.1371/journal.pone.0341037)
Supplement: S1 Table — Note: *Model I: fitted without predictor variables (null model); **Model II: fitted with only individual level variables (i.e., women’s education, women’s employment, household wealth, marital status, women’s age, parity, listening to radio, reading magazine, watching television, and currently breastfeeding); ***Model III: fitted with only regional variables (i.e., place of residence, and division); ****Model IV: fitted with all predictor variables (i.e., both individual and regional variables). aAIC: Akaike information criterion; bBIC: Bayesian information criterion. (PDF) [file pone.0341037.s001.pdf]

S1 Table. Results of Pseudo-R^2, log likelihood, AIC, and BIC to check the goodness of fit of the selected models. Note: *Model I: fitted without predictor variables (null model); **Model II: fitted with only individual level variables (i.e., women's education, women's employment, household wealth, marital status, women's age, parity, listening to radio, reading magazine, watching television, and currently breastfeeding); ***Model III: fitted with only regional variables (i.e., place of residence, and division); ****Model IV: fitted with all predictor variables (i.e., both individual and regional variables). [a]AIC: Akaike information criterion; [b]BIC: Bayesian information criterion

Model fitness	Model I* (null model)	Model II**	Model III***	Model IV****
Pseudo-R^2	0.0000	0.0578	0.0124	0.0626
log likelihood	-11796.208	-10734.087	-11650.450	-10679.300
[a]AIC	23598.416	21552.175	23354.901	21490.600
[b]BIC	23619.804	21850.129	23547.399	21958.814
