# Supplementary material for: Prevalence and determinants of underweight, overweight, and obesity among reproductive-aged Bangladeshi women: Evidence from Bangladesh Demographic and Health Survey 2022
Source: PLoS One. 2026 Feb 25;21(2):e0341037. doi: 10.1371/journal.pone.0341037 (PMC12935259; doi:10.1371/journal.pone.0341037)
Supplement: S2 Table — Note: aVIF: Variance Inflation Factor. (PDF) [file pone.0341037.s002.pdf]

S2 Table. Multicollinearity diagnosis result. Note: [a]VIF: Variance Inflation Factor.

Variable	[a]VIF	1/VIF
Women's education (vs. No education)		
Primary	2.37	0.422729
Secondary or higher	2.90	0.345365
Women's employment (vs. Not working)		
Professional/semi-professional	1.10	0.912088
Agricultural/manual	1.16	0.858726
Household wealth (vs. Poor)		
Middle	1.33	0.750231
Rich	1.76	0.567486
Women's age (vs. 15-24)		
25-34	1.98	0.503933
35-49	2.70	0.370729
Parity (vs. 0)		
1-4	2.07	0.483804
5 or more	2.08	0.480307
Reading magazine (vs. No)		
Yes	1.08	0.922807
Watching television (vs. No)		
Yes	1.17	0.855337
Currently breastfeeding (vs. No)		
Yes	1.35	0.743441
Place of residence (vs. Urban)		
Rural	1.27	0.785417
Division (vs. Dhaka)		
Barishal	1.25	0.800389
Chattogram	1.52	0.657373
Khulna	1.38	0.723963
Mymensingh	1.31	0.761896
Rajshahi	1.46	0.687000

Rangpur	1.42	0.705664
Sylhet	1.23	0.810025
Mean VIF	1.61	
