# Supplementary material for: Prevalence and determinants of underweight, overweight, and obesity among reproductive-aged Bangladeshi women: Evidence from Bangladesh Demographic and Health Survey 2022
Source: PLoS One. 2026 Feb 25;21(2):e0341037. doi: 10.1371/journal.pone.0341037 (PMC12935259; doi:10.1371/journal.pone.0341037)
Supplement: Attachment — Submitted filename: _Response to Reviwers [02 Dec 2025].docx [file pone.0341037.s004.docx]

| **Response to Reviewer 1 Comments** | | |
| --- | --- | --- |
| **1. Questions for General Evaluation** | **Reviewer’s Evaluation** | **Response and Revisions** |
| Is the manuscript technically sound, and do the data support the conclusions? | Partly | Thanks for this evaluation. Please see our point-by-point responses to the comments below. |
| Has the statistical analysis been performed appropriately and rigorously? | Yes |  |
| Have the authors made all data underlying the findings in their manuscript fully available? | No |  |
| Is the manuscript presented in an intelligible fashion and written in standard English? | Yes |  |
| **2. Point-by-point response to Comments and Suggestions for Authors** | | |
| **Comments 1:** [The manuscript, in its current form, may contain a critical data error that fundamentally undermines its findings and conclusions. The reported prevalence of obesity (79.08%) is epidemiologically implausible and may indicate a likely miscalculation or misclassification of BMI categories. The study itself cites the BDHS 2017-18, where the combined overweight/obesity rate for women was 45.60%. It is statistically impossible for the obesity rate alone to jump from a fraction of that 45.6% to 79.08% in just 4-5 years without a catastrophic societal shift, which has not occurred. This issue must be resolved before the manuscript can be considered for publication.]. | | |
| **Response 1**: Dear Reviewer, thank you for pointing this out. After re-checking the codes, we resolved the coding errors and revised the results when necessary. | | |
| **Comments 2:** [Abstract: The abstract selectively highlights "higher education levels" and "greater wealth" as key factors associated with overweight/obesity. However, the full results (Table 3) indicate that the strongest risk factor is older age (ARRR = 1.86 for 35-49 year-olds), followed by watching television (ARRR = 1.21), while the association with higher education is more modest (ARRR = 1.19). The narrative in the abstract should be revised to reflect the magnitude of the associations and prioritize the most influential risk factors identified by the analysis. The conclusion does not align wholly with the topic. The conclusion should reflect both the prevalence and the determinants. It introduces “the dual burden of malnutrition,” which is relevant, but that concept isn’t explicitly in your title — so it needs to be linked more clearly to your findings. 108 -109: the statement seem to suggest that the prevailing gender inequality in Bangladesh could be attributed to malnutrition in females compared to males. Is that the case?] | | |
| **Response 2:** Thank you for this feedback. Based on the updated analysis, the entire result and conclusion section was revised and incorporated according to the magnitude of the associations. Conclusion is now liked with the findings. | | |
| **Comments 3:** [Introduction: The introduction effectively establishes the public health significance of the double burden of malnutrition and provides a good rationale for focusing on women of reproductive age. The justification for using the most recent data is well-placed but could be more forcefully articulated by explicitly stating what changes (e.g., economic shifts, urbanization) since the last survey make this update crucial.] | | |
| **Response 3:** Thank you for your valuable feedback. We have revised the introduction to explicitly state the research gap and the rationale for conducting this study. | | |
| **Comments 4:** [The implausible BMI distribution (79.08% obese, 11.40% normal weight) must be investigated and corrected if an error was made.] | | |
| **Response 4:** Thank you for pointing this out. After re-checking the codes, we resolved the coding errors and revised the results when necessary. | | |
| **Comments 5: [**The analysis does not account for key behavioural determinants such as dietary patterns and physical activity levels, which are major limitations that should be explicitly acknowledged and discussed.**]** | | |
| **Response 5:** Thank you for the comment. However, we have already addressed the issues under the “Strengths and limitations” subsection in the manuscript. | | |
| **Comments 6: [**The entire discussion is based on a possibly incorrect prevalence estimate and must be entirely rewritten once the data error is fixed.] | | |
| **Response 6:** We have corrected the prevalence estimates and addressed the changes in our discussion section. However, some parts of the discussion were retained as the factors associated with underweight, overweight, and obesity remained similar to what we have found earlier. | | |
| **Comments 7:** [The discussion does not adequately address the limitations of the study, particularly the unmeasured confounding by diet and physical activity, and the cross-sectional nature of the data. Claims about public health implications are premature until the core findings are validated through data correction.] | | |
| **Response 7:** Thank you for the comment. However, we have already addressed the issues under the “Strengths and limitations” subsection in the manuscript. As mentioned above, we corrected the data and revised the concerned results and discussions accordingly. | | |
| **Comments 8:** [The conclusion should outline specific, actionable recommendations for public health policy and future research directions.] | | |
| **Response 8:** Thank you for your valuable suggestion. We have revised the conclusion to include clear, actionable recommendations for public health policy and outlined future research directions, as requested. | | |

| **Response to Reviewer 2 Comments** | | |
| --- | --- | --- |
| **1. Questions for General Evaluation** | **Reviewer’s Evaluation** | **Response and Revisions** |
| Is the manuscript technically sound, and do the data support the conclusions? | Yes | Thanks for this evaluation. Please see our point-by-point responses to the comments below. |
| Has the statistical analysis been performed appropriately and rigorously? | No |  |
| Have the authors made all data underlying the findings in their manuscript fully available? | Yes |  |
| Is the manuscript presented in an intelligible fashion and written in standard English? | Yes |  |
| **2. Point-by-point response to Comments and Suggestions for Authors** | | |
| **Comments 1:** [1. The reported BMI distribution (obesity = 79.08%, normal = 11.40%, overweight = 6.22% in 27,974 women) is implausible for BDHS 2022, even using Asian cut-offs. This likely reflects category misclassification. Please re-check: (i) BMI band recoding, (ii) possible label swap between overweight and obesity, and (iii) weighted percentages and denominators.]. | | |
| **Response 1**: Dear Reviewer, thank you for pointing this out. After re-checking the codes, we resolved the coding errors and revised the results when necessary. | | |
| **Comments 2:** [2. Present both Asian (23/27.5 kg/m²) and WHO (25/30 kg/m²) cut-off schemes in a sensitivity table or figure and discuss their effect on prevalence.] | | |
| **Response 2:** Thank you for pointing this out. We have addressed the comment accordingly. | | |
| **Comments 3:** [3. The text says “multilevel multinomial”, but Methods/Software say mlogit with svy, which is single level not multilevel. gsem is multilevel multinomial approach. So revise to “survey-weighted multinomial logistic regression”.] | | |
| **Response 3:** Thank you for your comment. We revised accordingly. | | |
| **Comments 4:** [4. In the univariable table, Rural = 0.37 (1.12–1.67) is impossible (CI not bracketing the point). There are several similar formatting/logic errors.] | | |
| **Response 4:** Thank you for pointing this out. We agree with your comment. Therefore, we addressed the comment accordingly. We have re-checked other results. | | |
| **Comments 5: [**5. The objective should avoid causal wording. Replace “risk factors” with “associated factors,” “determinants,” or “correlates.”**]** | | |
| **Response 5:** Thank you for your comment. We revised accordingly. | | |
| **Comments 6: [**6. The term “significant prevalence” is inappropriate unless a statistical comparison was performed. Use neutral wording such as “a high prevalence” or “a substantial prevalence.”] | | |
| **Response 6:** We agree with your comment. The conclusion section is now re-written based on the updated findings. | | |
| **Comments 7:** [7. In conclusion the phrase “risk identified” should be revised to “factors associated with” or “determinants identified.”] | | |
| **Response 7:** The term “risk” was replaced with “associated factors” where possible. The conclusion section was modified. | | |
| **Comments 8:** [8. Keep one decimal place consistently throughout tables, text, and figures for presenting percentages.] | | |
| **Response 8: T**hank you for your comment. We revised accordingly. | | |
| **Comments 9:** [9. The citation for BMI categorization (references no 31) appears incorrect. Verify that the cited reference truly describes the Asian or Bangladeshi BMI cut-off recommendation (should be: WHO Expert Consultation, Lancet 2004;364:157–63). Check other references accordingly.] | | |
| **Response 9:** Thank you for pointing this out. We have updated this reference and added the following:  *“WHO Expert Consultation. Appropriate body-mass index for Asian populations and its implications for policy and intervention strategies. Lancet. 2004 Jan 10;363(9403):157-63. doi: 10.1016/S0140-6736(03)15268-3. Erratum in: Lancet. 2004 Mar 13;363(9412):902. PMID: 14726171.”*  We have checked other references as well. | | |

Response to Academic Editor comments

Comment 1: [Journal Requirements:

When submitting your revision, we need you to address these additional requirements.

1. Please ensure that your manuscript meets PLOS ONE's style requirements, including those for file naming. The PLOS ONE style templates can be found at

<https://journals.plos.org/plosone/s/file?id=wjVg/PLOSOne_formatting_sample_main_body.pdf>and [https://journals.plos.org/plosone/s/file?id=ba62/PLOSOne_formatting_sample_title_authors_affiliations.pdf](https://track.editorialmanager.com/CL0/https:%2F%2Fjournals.plos.org%2Fplosone%2Fs%2Ffile%3Fid=ba62%2FPLOSOne_formatting_sample_title_authors_affiliations.pdf/1/010f019a2f3f8c10-d0354c3b-68ab-4e3a-b655-46301b83669a-000000/fIavBMj63qhnfYAfFth4r3LhcmsK9jcqAqxbZPHluTY=235)

If the reviewer comments include a recommendation to cite specific previously published works, please review and evaluate these publications to determine whether they are relevant and should be cited. There is no requirement to cite these works unless the editor has indicated otherwise.]

Response 1: Dear Academic Editor, thank you for your comments. We have addressed all the additional requirements accordingly.
